# Supplementary material for: Optimized libraries for CRISPR-Cas9 genetic screens with multiple modalities
Source: Nat Commun. 2018 Dec 21;9:5416. doi: 10.1038/s41467-018-07901-8 (PMC6303322; doi:10.1038/s41467-018-07901-8)
Supplement: Supplementary file 1 — Supplementary Information [file 41467_2018_7901_MOESM1_ESM.pdf]

Supplementary Information

Optimized libraries for CRISPR-Cas9 genetic screens with multiple modalities

Sanson & Hanna et al.

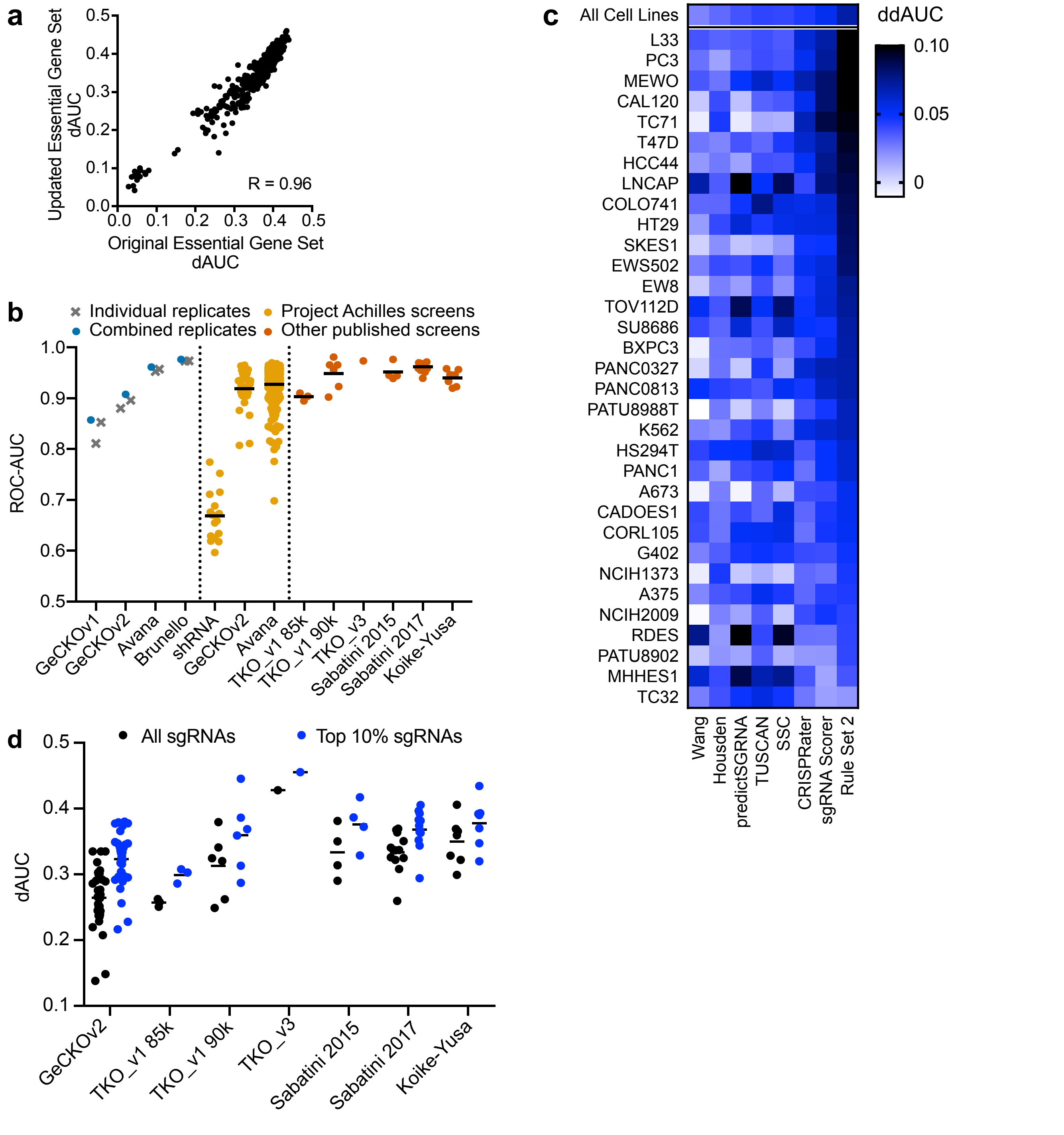

Supplementary Figure 1. Improved CRISPRko performance with the Brunello library. (a) Comparison of the dAUCs from Fig. 1c using the original and updated essential gene sets. Pearson correlation is reported. (b) Comparison of the ROC-AUC across different CRISPRko libraries. (c) Heat map displaying the the ddAUC of the GeCKOv2 library filtered by each scoring scheme relative to the unfiltered library, across the 33 cell lines screened in Project Achilles with GeCKOv2. (d) Comparison of dAUCs of published CRISPRko datasets and the same libraries filtered to examine only the top 10% of sgRNAs as picked by the scoring scheme used to design Brunello.

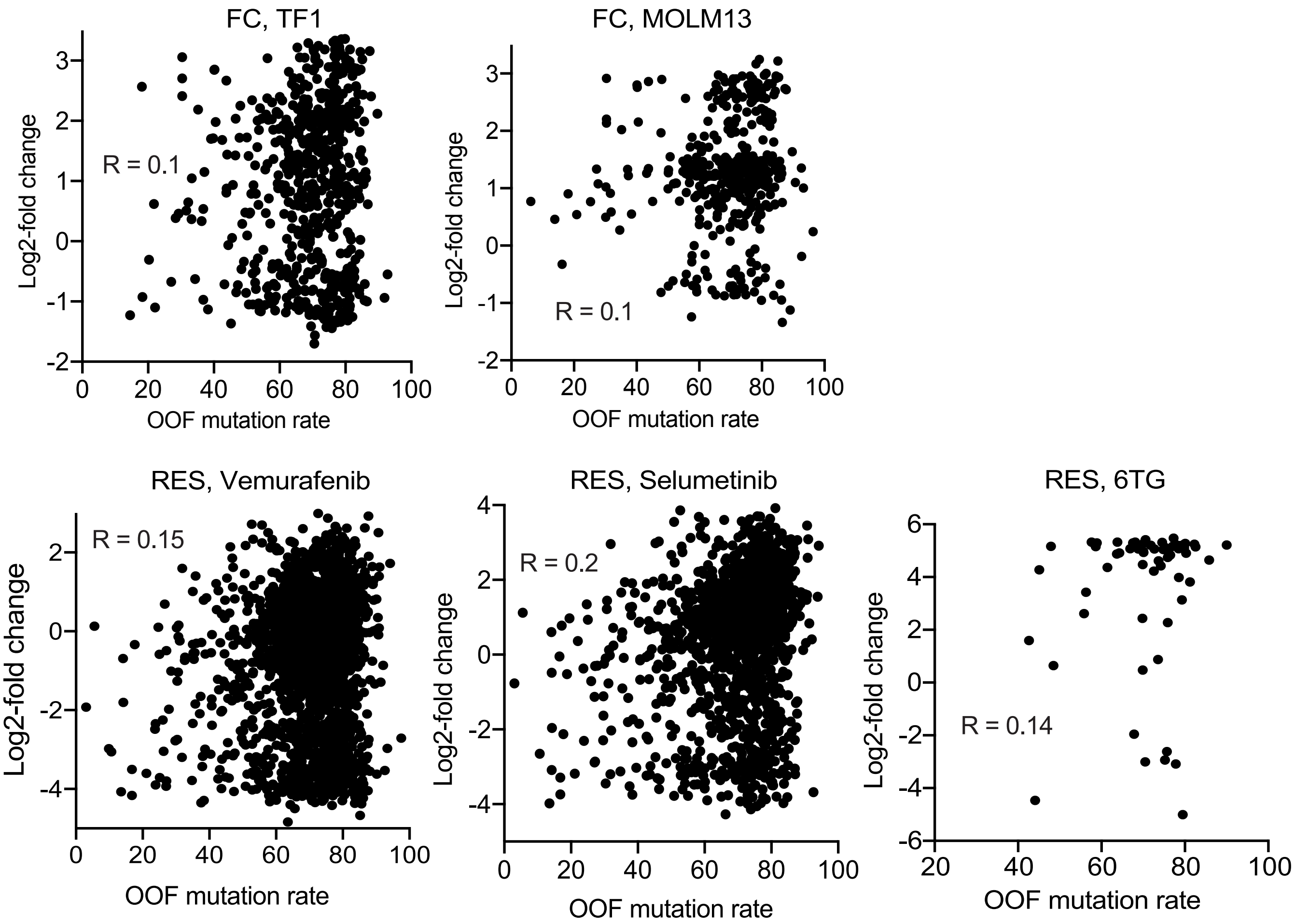

Supplementary Figure 2. Comparison of the measured log2-fold change values to the predicted out of frame (OOF) mutation rate for tiling libraries of sgRNAs assayed by flow cytometry (FC) in different cell lines or for resistance (RES) to different small molecules, including vemurafenib, selumetinib, and 6-thioguanine (6TG).

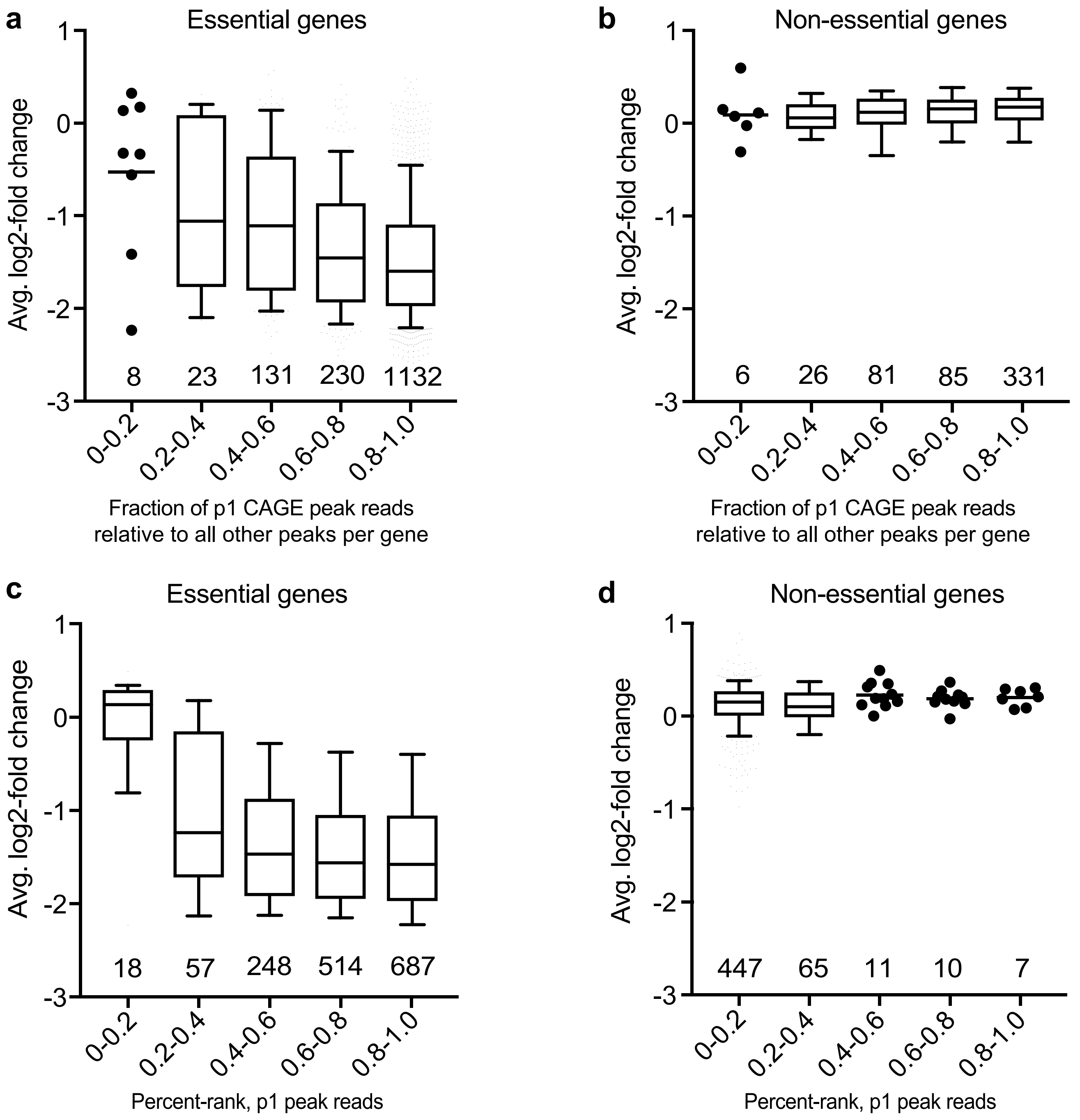

Supplementary Figure 3. Comparison of TSS confidence to the performance of Dolcetto Set A in A375 cells. (a) The fraction of CAGE peak reads assigned to the p1 promoter relative to all other peaks for that gene, compared to the activity of sgRNAs targeting essential genes (the bin 0.8 - 1.0 has the genes with the highest fraction of p1 promoter reads relative to all other promoters for that gene). The box represents the 25th, 50th, and 75th percentiles; whiskers show 10th and 90th percentiles. For the bin with 8 points the individual points are plotted and the mean indicated. The number of genes in each bin is indicated. (b) As in (a), for non-essential genes. (c) Analysis of the number of CAGE peak reads per gene, compared to the activity of sgRNAs targeting essential genes. Each gene was ranked by the number of CAGE peak reads corresponding to promoter p1, and then binned according to the percent rank (the bin 0.8 - 1.0 has the genes with the most p1 CAGE peak reads). The box represents the 25th, 50th, and 75th percentiles; whiskers show 10th and 90th percentiles. The number of genes in each bin is indicated. (d) As in (c) for non-essential genes.

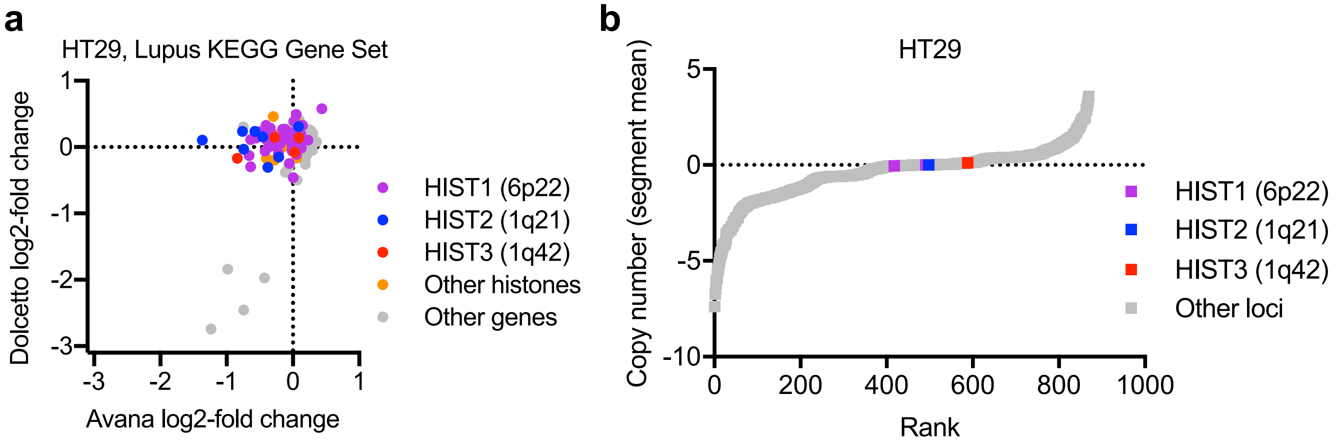

Supplementary Figure 4. Analysis of histone genes in HT29 cells. (a) Comparison of log2-fold change of genes from the KEGG Systemic Lupus Erythematosus gene set for Avana (CRISPRko) and Dolcetto (CRISPRi) in HT29 cells. (b) Segmented copy number (segment mean; log2-fold change from average) of genetic loci in HT29 cells.

Supplementary Table 1. Screening conditions used for published genome-wide CRISPRko libraries.

			Cas9 delivery			Library delivery				
Library	Reference	# of vectors	Vector	Selection	Addgene #	Vector	Selection	Addgene #	sgRNAs per gene	tracrRNA
GeCKOv1	Shalem 2014[1]	1	With library			lentiCRISPRv1	Puro	49535	3 - 4	tracr
GeCKOv2	Doench 2016 [2]	2	lentiCas9	Blast	52962	lentiGuide	Puro	52963	6	tracr
Avana	Doench 2016[2]	2	pLX_ 311-Cas9	Blast	96924	lentiGuide	Puro	52963	6	tracr
Brunello	Doench 2016[2]	2	pLX_ 311-Cas9	Blast	96924	lentiGuide	Puro	52963	4	tracr
Brunello (tracr-v2)	This paper	1	With library			lentiCRISPRv2	Puro	52961	4	tracr-v2
GeCKOv2 (Achilles)	Aguirre 2016[3]	2	lentiCas9	Blast	52962	lentiGuide	Puro	52963	6	tracr
Avana (Achilles)	Meyers 2017[4]	2	pLX_ 311-Cas9	Blast	96924	lentiGuide	Puro	52963	4	tracr
TKO_v1 90K	Hart 2015[5]	2	Cas9-2A-blast	Blast	N/A	pLKO.1	Puro	73311	6	tracr
TKO_v1 85K	Hart 2015[5]	2	Cas9-2A-blast	Blast	N/A	pLKO.1	Puro	73311	6	tracr
TKO_v3	Hart 2017[6]	1	With library			lentiCRISPRv2	Puro	52961	4	tracr
Sabetini 2015	Wang 2015[7]	1	With library			lentiCRISPRv1	Puro	49535	10	tracr
Sabetini 2017	Wang 2017[8]	1	With library			lentiCRISPRv1	Puro	49535	10	tracr
Koike-Yusa	Tzelepis 2016[9]	2	pKLV2- EF1a-Cas9Bsd-W	Blast	68343	pKLV2-U6gRNA5(BbsI)-PGKpuro2ABFP-W	Puro	67974	5	Modified tracr Chen 2013[10]

Supplementary Table 2. CRISPRi library design picking criteria. CRISPRi sgRNAs were picked in 12 rounds binned by position relative to TSS, off-target criteria and on-target criteria.

Picking Round	Position relative to TSS	Off-target criteria	On-target criteria - Rule Set2 score
1	+25 to +75	<=1 perfect match	>=0.2
2	0 to +100	<=1 perfect match	>=0.2
3	+175 to +250	<=1 perfect match	>=0.2
4	+25 to +75	<=5 perfect match	>=0.2
5	0 to +100	<=5 perfect match	>=0.2
6	+175 to +250	<=5 perfect match	>=0.2
7	+25 to +75	<=5 perfect match	>0
8	0 to +100	<=5 perfect match	>0
9	+175 to +250	<=5 perfect match	>0
10	-50 to +300	<=1 perfect match	>0
11	-50 to +300	<=1 perfect match	>0
12	-300 to +300	Any	Any

Supplementary Table 3. Screening conditions used for published genome-wide CRISPRi screens.

			Cas9 delivery			Library delivery				
**Library**	**Reference**	**# of vectors**	**Vector**	**Selection**	**Addgene #**	**Vector**	**Selection**	**Addgene #**	**sgRNAs per gene**	**tracrRNA**
Dolcetto Set A	This paper	2	pXPR_121 (KRAB-dCas9)	Blast	96918	pXPR_ 050	Puro	96925	3	tracr-v2
Dolcetto Set B	This paper	2	pXPR_121 (KRAB-dCas9)	Blast	96918	pXPR_ 050	Puro	96925	3	tracr-v2
hCRISPRi-v2	Horlbeck 2016[11]	2	pHR-SFFV-KRAB-dCas9-P2A -mCherry	mCherry	60954	pCRISPRia-v2	Puro	84832	10	Modified tracr Chen 2013[10]

Supplementary Table 4. CRISPRa library design picking criteria. CRISPRa sgRNAs were picked in 12 rounds binned by position relative to TSS, off-target criteria and on-target criteria.

Picking Round	Position relative to TSS	Off-target criteria	On-target criteria - Rule Set2 score
1	-150 to -75	<=1 perfect match	>=0.2
2	-200 to -25	<=1 perfect match	>=0.2
3	-250 to 0	<=1 perfect match	>=0.2
4	-150 to -75	<=5 perfect match	>=0.2
5	-200 to -25	<=5 perfect match	>=0.2
6	-250 to 0	<=5 perfect match	>=0.2
7	-150 to -75	<=5 perfect match	>0
8	-200 to -25	<=5 perfect match	>0
9	-250 to 0	<=5 perfect match	>0
10	-250 to 0	<=1 perfect match	>0
11	-300 to 0	<=1 perfect match	>0
12	-300 to +300	Any	Any

Supplementary Table 5. Screening conditions used for published genome-wide CRISPRa screens.

			Cas9 delivery				Activation domain delivery			Library delivery						
Library	Reference	# of vectors	Vector	Selection	Addgene #	Tethered domain	Vector	Selection	Addgene #	Recruited domain	Vector	Selection	Addgene #	sgRNAs per gene	tracrRNA	tracrRNA stem loops
Calabrese Set A (dCas9)	This paper	2	pXPR_118 (dCas9)	Hygro	113667	None	N/A			PP7-p65-HSF1	pXPR_ 502	Puro	96923	3	tracr-v14	2 MS2, 2 PP7
Calabrese Set A (dCas9 - VP64)	This paper	2	pXPR_109 (dCas9-VP64)	Blast	61425	VP64	N/A			PP7-p65-HSF1	pXPR_ 502	Puro	96923	3	tracr-v14	2 MS2, 2 PP7
Calabrese Set B (dCas9 - VP64)	This paper	2	pXPR_109 (dCas9-VP64)	Blast	61425	VP64	N/A			PP7-p65-HSF1	pXPR_ 502	Puro	96923	3	tracr-v14	2 MS2, 2 PP7
SAM (Zeo)	Konermann 2015[12]	3	Lenti dCAS-VP64 _Blast	Blast	61425	VP64	lenti MS2-P65-HSF1_Hygro	Hygro	89308	MS2-P65_HSF1	lenti sgRNA (MS2)_zeo	Zeo	61427	3	Modified tracr (2 MS2 aptamers)	2 MS2
SAM (Puro)	Konermann 2015[12]	3	Lenti dCAS-VP64 _Blast	Blast	61425	VP64	lenti MS2-P65-HSF1_Hygro	Hygro	89308	MS2-P65_HSF1	lenti sgRNA (MS2)_puro	Puro	73795	3	Modified tracr (2 MS2 aptamers)	2 MS2
hCRISPRa-v2	Horlbeck 2016[11]	3	dCas9-SunTag	Single-cell cloning	N/A	SunTag	scFV-VP64	GFP	N/A	N/A	pCRISPRia-v2	Puro	84832	10	Modified tracr Chen 2013[10]	N/A

SUPPLEMENTARY REFERENCES

1. Shalem, O. *et al.* Genome-scale CRISPR-Cas9 knockout screening in human cells. *Science* **343,** 84–87 (2014).
2. Doench, J. G. *et al.* Optimized sgRNA design to maximize activity and minimize off-target effects of CRISPR-Cas9. *Nat. Biotechnol.* **34,** 184–191 (2016).
3. Aguirre, A. J. *et al.* Genomic Copy Number Dictates a Gene-Independent Cell Response to CRISPR/Cas9 Targeting. *Cancer Discov.* **6,** 914–929 (2016).
4. Meyers, R. M. *et al.* Computational correction of copy number effect improves specificity of CRISPR–Cas9 essentiality screens in cancer cells. *Nat. Genet.* **49,** 1779 (2017).
5. Hart, T. *et al.* High-Resolution CRISPR Screens Reveal Fitness Genes and Genotype-Specific Cancer Liabilities. *Cell* **163,** 1515–1526 (2015).
6. Hart, T. *et al.* Evaluation and Design of Genome-Wide CRISPR/SpCas9 Knockout Screens. *G3* **7,** 2719–2727 (2017).
7. Wang, T. *et al.* Identification and characterization of essential genes in the human genome. *Science* **350,** 1096–1101 (2015).
8. Wang, T. *et al.* Gene Essentiality Profiling Reveals Gene Networks and Synthetic Lethal Interactions with Oncogenic Ras. *Cell* **168,** 890–903.e15 (2017).
9. Tzelepis, K. *et al.* A CRISPR Dropout Screen Identifies Genetic Vulnerabilities and Therapeutic Targets in Acute Myeloid Leukemia. *Cell Rep.* **17,** 1193–1205 (2016).
10. Chen, B. *et al.* Dynamic imaging of genomic loci in living human cells by an optimized CRISPR/Cas system. *Cell* **155,** 1479–1491 (2013).
11. Horlbeck, M. A. *et al.* Compact and highly active next-generation libraries for CRISPR-mediated gene repression and activation. *Elife* **5,** (2016).
12. Konermann, S. *et al.* Genome-scale transcriptional activation by an engineered CRISPR-Cas9 complex. *Nature* **517,** 583–588 (2015).
