# Supplementary material for: Optimized libraries for CRISPR-Cas9 genetic screens with multiple modalities
Source: Nat Commun. 2018 Dec 21;9:5416. doi: 10.1038/s41467-018-07901-8 (PMC6303322; doi:10.1038/s41467-018-07901-8)
Supplement: Supplementary file 3 — Description of Additional Supplementary Files [file 41467_2018_7901_MOESM3_ESM.pdf]

Title: Supplementary Data 1.
Description: Brunello CRISPR-knockout screening data. The “raw reads” tabs provide the counts of each sgRNA in the pDNA pool and after 3 weeks of growth; screens performed with the original tracrRNA and the modified tracr-v2 are provided on separate tabs. The “annotations” tab provides the mapping of sgRNAs to their gene target.

Title: Supplementary Data 2.
Description: Comparison of tracrRNAs. The “raw reads” tab provides the count of each sgRNA in each condition for the original tracr and the modified tracr. The “WTSequence” column gives the original sequence of the mismatched sgRNA. The “Mutation” column specifies the single-nucleotide mismatch that was created and “Mismatch Position” column specifies the position in the sgRNA at which this mismatch was created.

Title: Supplementary Data 3.
Description: Dolcetto CRISPRi screening data. The “raw reads” tabs provide the counts of each sgRNA in the pDNA pool and after 3 weeks of growth, labeled by cell line. The “annotations” tabs provide the mapping of sgRNAs to their gene target.

Title: Supplementary Data 4.
Description: Annotation of histone gene depletion and copy number. The “Lupus KEGG Gene Set” and “histone gene set” tabs include the genes in the KEGG Systemic Lupus Erythematosus gene set and the HGNC histone gene set, respectively. The “log-fold change” tabs provide the average log2-fold change values by gene for A375 cells in Brunello and Dolcetto (both sets A and B) and for HT29 cells in Avana and Dolcetto (both sets A and B), as well as the karyotype band, chromosome location, and gene start and end position. The “copy number” tabs provide segmented copy number data from Project Achilles for A375 and HT29 cells.

Title: Supplementary Data 5.
Description: Calabrese CRISPRa screening data. The “raw reads” tabs provide the counts of each sgRNA in each condition. The “annotations” tabs provide the mapping of sgRNAs to their gene target. The “analysis” tabs provide the average log2-fold-change (LFC) values for all sgRNAs targeting a gene (Column B) and the p-value determined by the hypergeometric distribution (Column C).

Title: Supplementary Data 6.
Description: Secondary CRISPRa screen for vemurafenib resistance in A375 cells with a customized follow-up library.

Title: Supplementary Data 7.
Description: Secondary CRISPRa screen for MEK inhibitor resistance in MelJuSo cells with a customized follow-up library.
